# Gain of chromosome 21 in hematological malignancies: lessons from studying leukemia in children with Down syndrome

**DOI:** 10.1038/s41375-020-0854-5

**Published:** 2020-05-20

**Authors:** Anouchka P. Laurent, Rishi S. Kotecha, Sébastien Malinge

**Affiliations:** 1 INSERM U1170, Gustave Roussy Institute, Université Paris Saclay, Villejuif, France; 2 Université Paris Diderot, Paris, France; 3 School of Pharmacy and Biomedical Sciences, Curtin University, Perth, Western Australia Australia; 4 Department of Clinical Haematology, Oncology and Bone Marrow Transplantation, Perth Children’s Hospital, Perth, Western Australia Australia; 5 Telethon Kids Cancer Centre, Telethon Kids Institute, University of Western Australia, Perth, Western Australia Australia

**Keywords:** Haematological cancer, Cancer genetics

## Abstract

Structural and numerical alterations of chromosome 21 are extremely common in hematological malignancies. While the functional impact of chimeric transcripts from fused chromosome 21 genes such as TEL-AML1, AML1-ETO, or FUS-ERG have been extensively studied, the role of gain of chromosome 21 remains largely unknown. Gain of chromosome 21 is a frequently occurring aberration in several types of acute leukemia and can be found in up to 35% of cases. Children with Down syndrome (DS), who harbor constitutive trisomy 21, highlight the link between gain of chromosome 21 and leukemogenesis, with an increased risk of developing acute leukemia compared with other children. Clinical outcomes for DS-associated leukemia have improved over the years through the development of uniform treatment protocols facilitated by international cooperative groups. The genetic landscape has also recently been characterized, providing an insight into the molecular pathogenesis underlying DS-associated leukemia. These studies emphasize the key role of trisomy 21 in priming a developmental stage and cellular context susceptible to transformation, and have unveiled its cooperative function with additional genetic events that occur during leukemia progression. Here, using DS-leukemia as a paradigm, we aim to integrate our current understanding of the role of trisomy 21, of critical dosage-sensitive chromosome 21 genes, and of associated mechanisms underlying the development of hematological malignancies. This review will pave the way for future investigations on the broad impact of gain of chromosome 21 in hematological cancer, with a view to discovering new vulnerabilities and develop novel targeted therapies to improve long term outcomes for DS and non-DS patients.

## Introduction

Copy Number Variation (CNV), i.e. gains or losses of entire chromosomes or of specific genomic regions, are hallmarks of cancer. Understanding the impact of CNVs in tumor development is challenging since they can sometimes alter the dosage of hundreds or even thousands of genes simultaneously, thereby modifying mRNA and protein abundance, ultimately impacting cellular fitness. In hematological cancer, numerous CNVs are found and they are markedly different to those found in solid tumors. Gain of chromosome 21 (+21) is one of the most frequent CNVs observed in hematological malignancies [1, 2]. Using large cohorts, it has been shown that +21 is rarely seen in solid tumors and that trisomy 21, the most common type of +21, is found in nearly all subtypes of hematological malignancies, ranging from 2.2% in chronic lymphocytic leukemia (CLL) to nearly 15% in acute lymphoblastic leukemia (ALL) (Mitelman database, available at https://mitelmandatabase.isb-cgc.org). It is rarely observed as a sole cytogenetic abnormality and its prognostic value varies depending on the cohort analyzed and the type of hematological malignancy [1, 3]. Children with acute megakaryoblastic leukemia (AMKL) and B-cell precursor ALL (B-ALL) harbor +21 most frequently, occurring in approximately one-third of cases [4, 5]. To date, reasons for such an association remain elusive, but strongly suggest that somatic +21 is clonally selected during leukemia development and that megakaryocytic and B-cell progenitor/precursors are profoundly susceptible to the increased dosage of chromosome 21 genes.

Individuals with Down syndrome (DS) harbor constitutive trisomy 21 [6] and are predisposed to childhood acute leukemia [7]. Constitutive trisomy 21 is the most common cytogenetic abnormality seen at birth (1 in 700–1000 newborns), and predominantly results from nondisjunction of chromosome 21 during meiosis (95%) [8–10]. Although this mechanism is different to somatic gain of chromosome 21, studying the predisposing and leukemia promoting role of trisomy 21 in DS human specimens, cell lines and murine models has been instrumental in undertanding the role of +21 alone and in cooperation with other secondary genetic alterations. Evidence indicates that trisomy 21 (or gain of chromosome 21), regardless of whether constitutive or acquired, is a promoting event in hematological malignancies.

This review will present a snapshot of our knowledge on DS-associated leukemia, integrating studies addressing clinical features, therapy and molecular mechanisms of leukemogenesis, and will consider them in parallel to other hematological malignancies harboring somatic +21 with the view to emphasize similarities and differences at the clinical and biological level.

## Clinical features, therapy, and outcome of DS-associated leukemia

### Clinical features

Common clinical features seen in individuals with DS are intellectual disability, congenital heart defects, Alzheimer’s disease and immunodeficiency, among many others [8]. Incidence and severity of these phenotypes can vary due to trisomy 21 itself (complete or segmental trisomy) and/or potential modifier genes that remain elusive to date. Individuals with DS have a unique pattern of malignancies compared with the general population, characterized by a decreased incidence of solid tumors in adults and predisposition to leukemia during childhood, predominantly myeloid leukemia (ML–DS, >100-fold increased risk), which has a high prevalence of AMKL, and ALL (DS–ALL) [7].

Indicative of an intrinsic effect of trisomy 21 on hematopoiesis, almost all neonates with DS have quantitative and/or qualitative disorders of the myeloid compartment such as macrocytosis, dysplastic platelets, leukocytosis, and on average 4% blasts in the peripheral blood [11]. Approximately 10% develop transient myeloproliferative disorder (TMD), which is classically defined by the presence of megakaryoblasts in the peripheral blood, liver and bone marrow [12]. TMD spontaneously resolves within the first months of life, suggesting that mechanisms regulated by the fetal microenvironment maintain perturbed hematopoiesis. Up to 30% of children with DS who have classically defined TMD develop ML–DS before 5 years of age. This occurs through stepwise pathogenesis with an incremental acquisition of genetic alterations (including GATA1 mutations, see below). However, ~20% of neonates with DS have been identified as having ‘silent TMD,’ defined by a peripheral blast count of ≤10% and detection of a *GATA1* gene mutation by next generation sequencing [11]. This discovery has implications for the population at risk of transforming to ML–DS. Children with ML–DS have remarkably good prognosis compared with non-DS children with acute myeloid leukemia (AML), with 5-years overall and event-free survival of 89–93% and 87–90% respectively [13, 14].

Children with DS have a 27-fold increased risk of developing ALL [7]. A recent study has emphasized that these are almost exclusively B-cell phenotype, with T-cell ALL identified in only 5 of 653 children with DS [15]. In contrast to ML–DS, children with DS–ALL have an inferior outcome compared with non-DS children with ALL due to higher relapse rates, increased risk of infection, treatment-related mortality and induction failure [15, 16].

### Therapy and outcome

Significant progress has been made in the treatment of children with DS and leukemia, with outcomes summarized in Table 1. Several studies have prospectively collected and published data on TMD in neonates with DS. These studies have been instrumental in identifying that the majority of patients undergo spontaneous remission, demonstrated a clear benefit for treating babies with high-risk features with low-dose cytarabine (which reduces TMD-related mortality but does not prevent progression to ML–DS) and indicated that persistence of minimal residual disease (MRD) can be used to predict risk for developing ML–DS [17–21]. The next generation of trials should seek to uniformly define high-risk criteria for therapy, identify the optimal dose and schedule for cytarabine treatment and identify the molecular mechanisms that underpin progression to ML–DS. Given the rarity of the disease, consideration should be given to a unified international protocol, which will provide uniformity of outcomes and permit a greater number of questions to be answered.

**Table 1:** Summary of clinical trials for children with Down syndrome and leukemia.

**Transient myeloproliferative disorder (TMD)**										
Group	Study	Year	Evaluable patients		SR (%)	TMD-related death (%)	Developed ML–DS (%)	EFS (%)	OS (%)	Reference
POG	9481	1996–1999	47		89.4	–	17.0	–	–	[18]
COG	2971	1999–2004	135		78.5	10.4	15.6	57 (3-year)	77 (3-year)	[20]
BFM	AML–BFM studies	1993–2006	146		66.4	8.9	19.9	63 (5-year)	85 (5-year)	[19]
BFM/DCOG	TMD07	2007–2015	102		–	4.9	16.7	72 (5-year)	91 (5-year)	[21]

**Myeloid leukemia (ML–DS)**										
Group	Study	Year	Evaluable patients	Prior TMD	CR	TRM (%)	Relapses (%)	EFS (%)	OS (%)	Reference
COG	2971	1999–2003	132	57	91/108	2.3	–	79 (5-year)	84 (5-year)	[26]
Japanese Childhood AML Cooperative Study Group	AML99	2000–2004	72	9	70/72	1.4	12.5	83.3 (4-year)	83.7 (4-year)	[24]
JCCLSG	AML 9805	1998–2006	24	7	21/24	12.5	4.2	82.6 (5-year)	87.5 (5-year)	[25]
JPLSG	AML-D05	2008–2010	72	35	69/72	1.4	13.9	83.3 (3-year)	87.5 (3-year)	[27]
COG	AAML0431	2007–2011	204	63	177/202	1.0	6.9	89.9 (5-year)	93.0 (5-year)	[13]
BFM/DCOG/NOPHO	ML–DS 2006	2006–2015	170	43	–	2.9	5.3	87 (5-year)	89 (5-year)	[14]

**Acute lymphoblastic leukemia (DS–ALL**)^a^										
Group	Study	Year	Evaluable patients		CR	TRM (%)	Relapses (%)	EFS (%)	OS (%)	Reference
French Leukaemia Registry	Registry	1990–2008	92		88/92	9.8	26.1	64.1 (5-year)	73.6 (5-year)	[143]
PPLLSG	ALL IC-BFM 2002	2003–2010	41		–	9.8	19.5	–	86 (5-year)	[36]
DFCI	DFCI 00-001 and DFCI 05-001	2000–2011	38		38/38	0.0	10.5	91 (5-year)	97 (5-year)	[37]
CCG	CCG 1991	2000–2005	75^b^		–	1.3	9.3	86.9 (10-year)	91.1 (10-year)	[38]

*TMD* transient myeloproliferative disorder, *ML–DS* myeloid leukemia associated with Down syndrome, *DS-ALL* acute lymphoblastic leukemia associated with Down syndrome, *SR* spontaneous remission, *EFS* event-free survival, *OS* overall survival, *CR* complete remission, *TRM* treatment-related mortality, *POG* Pediatric Oncology Group, *COG* Children’s Oncology Group, *BFM* Berlin–Frankfurt–Münster study group, *DCOG* Dutch Childhood Oncology Group, *JCCLSG* Japanese Children’s Cancer and Leukemia Study Group, *JPLSG* Japanese Pediatric Leukemia/Lymphoma Study Group, *NOPHO* Nordic Society of Pediatric Hematology and Oncology, *PPLLSG* Polish Pediatric Leukemia and Lymphoma Study Group, *DFCI* Dana-Farber Cancer Institute, *CCG* Children’s Cancer Group.

^a^Trials published subsequent to Lee et al. [35].

^b^Randomized patients.

Prior to the 1980s, children with ML–DS were undertreated resulting in a high rate of treatment failure [22]. ML–DS patients were subsequently registered on protocols used for non-DS AML. Increased survival was evident, with lower rates of induction failure and relapse, however treatment-related mortality was more frequent resulting in protocol adaptation to dose-reduce therapy or prolong the interval between chemotherapy courses [23]. Over the last 20 years, children have been enrolled onto uniform ML–DS specific protocols (Table 1) [13, 14, 24–27]. These studies have been instrumental in highlighting the benefit for reduced-intensity ML–DS specific protocols, with outcomes for children with ML–DS significantly better than non-DS AML. Due to concerns regarding increased treatment-related toxicity in children with ML–DS [28–30], sequential protocols successfully reduced cumulative exposure to several agents including daunorubicin, etoposide, and intrathecal cytarabine without impacting on overall outcome [13, 14, 26, 27]. High-dose cytarabine was established as an important component of therapy, with early administration leading to improved outcomes [13] and subsequent attempts to omit high-dose cytarabine in standard-risk patients resulting in significantly lower event-free survival [31]. Several studies have identified older age as an unfavorable independent prognostic feature [26, 27, 32]. ML–DS diagnosed in children over 4 years of age has been shown to lack *GATA1* mutations and has a cytogenetic profile more akin to children with sporadic AML [33]. Given that biologically the disease appears to represent sporadic AML occurring in children with DS rather than ML–DS, undertreatment on the less intensive ML–DS protocols may account for the adverse prognosis in this age group. As such, it has been suggested that children within this older age group may benefit from more intensive therapy corresponding to that given to children with sporadic AML [33]. Monosomy 7 [24], gain of chromosome 8 [14], normal karyotype and high white blood cell count ≥20 × 10^9^/l at presentation [32] have also been identified in individual reports as independent variables associated with an inferior outcome. Detection of MRD after induction therapy using both deep sequencing of GATA1 and flow cytometric methods has been identified as a significant prognostic factor for predicting relapse [34], with current trials implementing risk-adapted therapy according to MRD response (NCT02521493; jRCTs041190047). Future studies should prospectively establish whether additional clinical and biological features can be utilized in addition to MRD response assessment for risk-stratification, which may help further reduce therapy in low-risk patients without compromising outcome and enable treatment intensification for high-risk patients to prevent relapse.

Children with DS–ALL are treated on standard ALL chemotherapeutic protocols [35–38]. Similar to ML–DS, sequential treatment protocols have identified that children with DS–ALL are more prone to treatment-related toxicity due to heightened sensitivity to chemotherapeutic agents, particularly methotrexate, and infectious complications, further contributing to their inferior outcome [16]. Consequently, this has resulted in modification of treatment to reduce intensity and implementation of intensified supportive care measures for children with DS–ALL [39, 40]. We are rapidly approaching a therapeutic plateau to which we can intensify conventional chemotherapeutic agents in order to balance the equilibrium between relapse and treatment-related toxicity, indicating the need to investigate novel agents in children with DS–ALL. Integration of the bispecific T-cell engager, blinatumomab, into the treatment backbone is being investigated in the current upfront COG (NCT03914625) and AIEOP-BFM (NCT03643276) studies and children with high-risk DS–ALL who are MRD positive at the end of consolidation are eligible for the single arm, phase 2 study of tisagenlecleucel (NCT03876769).

Outcomes for relapsed/refractory leukemia in children with DS are extremely poor [13, 41, 42]. Stem cell transplantation has been associated with high rates of relapse and treatment-related mortality in children with DS [43–45]. CAR T-cell therapy may provide an alternative option to stem cell transplantation in this setting. Children with relapsed/refractory DS–ALL have been included in studies using tisagenlecleucel, with preliminary results identifying comparable safety and efficacy to children without DS [46]. Such findings highlight the importance of including children with DS in studies of new agents for the treatment of leukemia to identify innovative approaches to reduce rates of relapse and treat relapsed disease [47]. Novel therapeutic strategies targeting the somatic events found in DS leukemia or the mechanisms altered by trisomy 21 as the initiating event in DS leukemogenesis should also be explored, which ultimately may be applicable to a broad spectrum of hematological malignancies that present with a similar genetic background.

## Genetic landscape of DS-associated leukemia

Several studies have recently reported on the genetic landscape of DS-associated leukemia, providing new insights into leukemia development. Ongoing functional characterization of secondary alterations that have been identified may shed light on novel actionable targets that could be therapeutically exploited.

### The multi-step pathogenesis of ML–DS development

Development of ML–DS, from predisposing trisomy 21 to pre-leukemia TMD to frank ML–DS, has been considered a model of sequential acquisition of secondary alterations for many years.

Discovery of *GATA1* mutations in nearly all patients with TMD and ML–DS was the first major breakthrough in our understanding of ML–DS [48–50]. The transcription factor GATA1 is a master regulator of erythroid and megakaryocytic lineages. In TMD/ML–DS, *GATA1* mutations are almost exclusively localized in exon 2 (97% of cases) and are predominantly insertions, deletions or duplications [51]. All *GATA1* mutations lead to the appearance of a premature stop codon. Consequently, a ‘short’ GATA1 isoform (GATA1s), truncated from its amino-terminal transactivation domain is expressed. Identical *GATA1* mutations are seen in TMD and at progression to ML–DS, regardless of whether they are present in the major clone [51–55]. The majority of these clones have leukemia-initiating and self-renewal potential [56, 57]. To date, the type of *GATA1* mutation, the level of *GATA1s* expression, the TMD blast karyotype and the size of dominant GATA1-bearing clones have not been shown to predict progression from TMD to ML–DS [51, 54, 55]. However, features identified as being predictive of progression include the persistence of immunophenotypic TMD blasts (>0.1%) or detection of *GATA1* mutations by quantitative PCR using patient-specific oligonucleotides at week 12 and the presence of pleural effusions at diagnosis of TMD [19, 21].

**Fig. 1:**
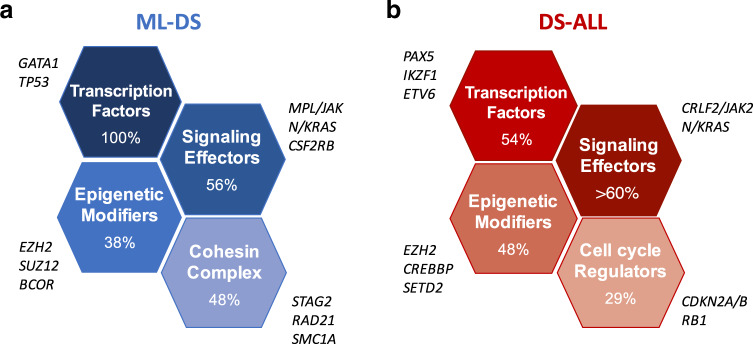
Somatic alterations found in DS-associated leukemia. **a** Four of the most common types of alteration found in ML–DS, in addition to constitutive trisomy 21. **b** Four of the most common types of alteration found in DS–ALL, in addition to constitutive trisomy 21.

Clonal evolution from TMD to ML–DS has been linked to acquisition of several secondary chromosomal and genetic alterations. There is a lower incidence of CNVs and chromosomal translocations in ML–DS compared with non-DS children with AML; the most common cytogenetic alterations being +8, dup(1q), and a fourth chromosome 21 [4, 5]. Next generation sequencing experiments have uncovered the mutational spectrum of TMD/ML–DS [54, 55, 58], revealing *GATA1* mutations as the only somatic mutations seen in TMD in most cases. During progression to ML–DS, two to five additional mutations are found. Among them, the most frequently altered genes encode signaling effectors (JAK1/2/3, MPL, RAS) in 56% of cases, members of the cohesin complex or associated components (STAG2, RAD21, SMC1A) in 48%, and epigenetic regulators (EZH2, SUZ12, and BCOR) in 38% (Fig. 1a). A novel clonal gain of function mutation affecting the *CSF2RB* gene, encoding the common beta chain of the IL3, IL5, and CSF cytokine receptors, was recently identified in almost 5% of patients with ML–DS and was mutually exclusive with *JAK1-3*, *MPL* or *RAS* mutations [55]. In ML–DS, somatic variants in signaling effectors and cohesin complex components are likely clonal and frequently co-occur, indicative of a potential cooperative effect in trisomic *GATA1s*-expressing hematopoietic progenitors [54, 55]. To date, nearly 75–80% of patients with ML–DS have been shown to harbor secondary alterations, emphasizing the model of progression from the pre-leukemic TMD stage. The molecular bases of clonal evolution for the remainder remains obscure to date.

Numerous in vitro and in vivo assays have reported on the role of somatic alterations found in TMD/ML–DS. *GATA1s* expression has been shown to promote megakaryoblastic progenitor expansion during fetal life but does not to lead to ML–DS [59]. Most of the somatic alterations found in ML–DS are shared with non-DS leukemia, and have been shown to promote self-renewal, differentiation blockade, proliferation and survival of hematopoietic stem cells (HSC) and/or myeloid progenitor populations [60, 61]. To assess leukemia progression from *GATA1s*-expressing cells without trisomy 21, models of oncogenic cooperation have been developed (retroviral insertional mutagenesis, ectopic expression and ‘loss of function screening’ using a CRISPR/Cas9 strategy and transgenic models) [55, 62, 63]. On average, 2.7 alterations on top of *Gata1s* expression was sufficient for the development of an erythro-megakaryoblastic leukemia in vivo, resembling ML–DS but strongly biased toward the erythroid lineage (CD117+Ter119+ population) [55]. The most common alterations found in these recipients were variants found in signaling effectors (73.7%) and epigenetic regulators (79%). The loss of function mutations in genes encoding components of the cohesin complex were underrepresented (16% in murine recipients vs 48% in patients with ML–DS). Whether this is due to human versus murine differences, or indicative of the lack of trisomy 21 in this model remains unknown.

In summary, observations in humans and mice have emphasized the essential role of trisomy 21, the fetal hematopoietic context of *GATA1s* expression and the requirement of additional genetic events predominantly affecting signaling effectors, epigenetic regulators and cohesin complex components to drive progression toward ML–DS.

### Somatic alterations found in DS–ALL

DS–ALL is a heterogeneous subtype of B-ALL that has a distinct cytogenetic profile compared with other types of childhood B-ALL (non-DS–ALL). Approximately 40% of DS–ALL cases have a normal karyotype other than the constitutional trisomy 21, 8–10% express the ETV6-RUNX1 fusion transcript, while 9–11% have high hyperdiploidy (HeH) [4, 15]. The most frequent CNV in DS–ALL is gain of chromosome X (38% vs 21% in non-DS–ALL); a twofold increase of del(9p) is also found in DS–ALL [4]. DS–ALL also has a higher proportion of rearrangements affecting the *CRLF2* locus (~50% in DS–ALL vs 4–5% in non-DS–ALL), with interstitial deletion of the pseudoautosomal region PAR1 (that fuses *CRLF2* to the first non-coding exon of *P2RY8*) or chromosomal translocation to the immunoglobulin heavy chain (IgH) locus [64–66]. These rearrangements lead to overexpression of the *CRLF2* gene, encoding a protein that heterodimerizes with IL7RA to form the thymic stromal lymphopoietin receptor. To date, there are no fusion proteins uniquely found in DS–ALL [67, 68].

At the gene level, *JAK2* activating mutations are present in 20–40% of patients with DS–ALL and predominantly affect the arginine 683 residue, located in the pseudokinase domain of JAK2 [69] (Fig. 1b). These *JAK2* mutations are virtually always found in *CRLF2*-overexpressing cases, indicative of a mechanism of oncogenic cooperation, as shown in experimental models [65, 66, 70]. In non-*JAK2* mutated or non-*CRLF2*-overexpressing cases, gain of function mutations are found in *CRLF2* or *IL7RA* genes, strongly implicating cytokine signaling as a major transforming process in DS–ALL [66, 70, 71]. Activating mutations affecting other signaling effectors, such as NRAS, KRAS, KIT, FLT3, and PTPN11, are also frequently found and are often mutually exclusive with *JAK2* mutations, highlighting the high incidence (>60%) of constitutively active signaling pathways in DS–ALL [67, 72]. In line with these findings, a recent study revealed enrichment of a Philadelphia-like transcriptional signature in DS–ALL [68]. Other common genetic abnormalities found in DS–ALL affect genes encoding cell cycle regulators (CDKN2A/B, RB1), transcription factors (PAX5, IKZF1, and ETV6) and epigenetic modifiers (EZH2, SETD2, and CREBBP) (Fig. 1b). Of interest, a recent genome-wide association meta-analysis explored inherited genetic susceptibility to ALL in children with DS, highlighting increased germline penetrance of the rs3731249 *CDKN2A* risk locus (9p21.3) [73].

To our knowledge, there is only one in vivo model of DS–ALL [74] and several other models assessing the cooperation between secondary mutations commonly found in DS–ALL. One study hypothesized that loss of *USP9X* (seen in 4/17 *CRLF2*-rearranged cases), which encodes a deubiquitinase known to stabilize activated JAK2, may naturally buffer the toxic effect of JAK/STAT signaling hyperactivation in DS–ALL [67]. In addition, activating mutations in signaling effectors have been shown to functionally cooperate with loss of *Ink4a/Arf* and *Pax5* alterations to drive B-cell leukemia development in vivo [75, 76].

Together, these studies unravel the high genetic complexity observed in DS–ALL, raising critical questions regarding the role of somatic alterations in the context of trisomy 21 in leukemia development. New models of DS–ALL will be required in the future to better understand the impact of trisomy 21, the mechanisms of cooperation and develop novel targeted therapies to improve outcome.

## Constitutive trisomy 21 as a ‘priming’ and cooperating event

Constitutive trisomy 21 (T21) results in altered hematopoiesis affecting many lineages, is developmental stage selective, but is not sufficient to lead to leukemia. Studying embryonic, fetal and adult hematopoiesis in individuals with DS and from genetically engineered models has unraveled the cellular and molecular bases of DS-leukemogenesis, and allowed identification of chromosome 21 dosage-sensitive genes.

### Insights from human DS fetal hematopoiesis

Compared with gestation-matched controls, analyses of second trimester human DS fetal livers revealed that trisomy 21 disturbs fetal hematopoiesis [77–79]. There is an increased proportion of HSC and of megakaryocyte-erythroid progenitors (MEP) in T21 fetal livers, at the expense of granulocyte-monocyte progenitors (GMP) and committed B progenitors. This bias toward the erythro-megakaryocytic lineage has been confirmed by clonogenic assays, liquid cultures, transplantation assays in immunodeficient mice and transcriptional analyses. A recent study showed that engineered *GATA1s* expression in human fetal liver HSC cooperates with trisomy 21 to promote blast and megakaryocyte expansion in xenotransplantation models [80]. The marked impairment of B lymphopoiesis in T21 fetal livers is also associated with a reduced proportion of both preproB (CD34+CD19+CD10−) and proB (CD34+CD19+CD10+) cells, and a decreased expression of early lymphoid or key lymphoid specific genes (such as *IKZF1*, *FLT3, PAX5*, and *IL7RA*) in early lymphoid progenitors [79]. Importantly, this reduced B-cell progenitor compartment is also seen in T21 fetal bone marrow, and is associated with impaired B-cell differentiation potential in vitro that may be linked to inflammatory signatures driven by the trisomic microenvironment [81].

This perturbed fetal hematopoiesis may provide insight into the cellular context of susceptibility for transformation of the megakaryocytic and B-cell lineages. However, several questions remain regarding the dynamic changes of these phenotypes during gestation before and after the second trimester, if they are maintained postnatally and throughout the life of individuals with DS, and how they may be affected by their microenvironment (yolk-sac, fetal liver and fetal and adult bone marrow).

### Insights from human DS-induced pluripotent stem cells

Through the establishment of DS-derived induced pluripotent stem cells (iPSCs), independent studies have shown that trisomy 21 alone significantly accelerates the early stages of hematopoiesis [82–85]. Regardless of whether primitive (yolk-sac type) or definitive (fetal liver type) hematopoiesis was induced, all groups reported an increase in the clonogenic potential of erythroid progenitors and enhanced erythroid differentiation. However, in contrast to primitive-like hematopoietic progenitors [83], mimicking fetal hematopoiesis from trisomic iPSCs led to multi-lineage expansion compared with disomic cells, characterized by significant expansion of myeloid and megakaryocytic progenitors in colony-forming unit (CFU) assays [82, 84, 85]. These observations were confirmed by silencing one chromosome 21 in DS-derived iPSCs using an inducible XIST strategy [84]. Extensive analyses of trisomic/GATA1s iPSCs, established from primary patient TMD samples or engineered by genome editing, have shown that *GATA1s* expression correlates with defective embryonic erythropoiesis and confers a strong bias toward the myelo-megakaryocytic compartment, indicating that both events cooperate in the development of TMD during fetal life [85, 86].

A recent study reported that trisomic CD34+ hematopoietic progenitors derived from embryoid bodies also have decreased ability to generate CD19+ B cells compared with isogenic controls [87]. This phenotype has been linked to reduced expression of endothelin signaling, and provides potential explanation for the impaired B-cell differentiation in human trisomic fetal livers [79], and for the decreased number of circulating B cells in individuals with DS [88].

Overall, these findings suggest that trisomy 21 perturbs early hematopoiesis to create permissive cellular contexts, for the development of both ML–DS and DS–ALL, through the acquisition of additional genetic alterations.

### Partially trisomic mice as models to study DS-leukemia

In the mouse genome, syngeneic regions of human chromosome 21 (Hsa21) are located on the three murine chromosomes (Mmu) 16, 17, and 10. Several partially trisomic murine models, containing some or all these syngeneic regions, have been used to study the impact of trisomy 21 on hematopoiesis, alone or in cooperation with *GATA1s* expression. The phenotypes of these models have been extensively reviewed [12, 89]. Here, we will briefly describe the phenotypes of murine transgenic DS models that have been used to define a minimal trisomic region and identify Hsa21 dosage-sensitive genes.

Ts65Dn is the most commonly used model to understand the phenotypes associated with DS [90]. This strain contains 104 trisomic genes, all located on Mmu16. Ts65Dn mice display an increased number of HSC and GMP, as well as decreased MEP during adulthood [91]. These mice develop progressive myeloproliferative disorder characterized by megakaryocytic hyperplasia, thrombocytosis, and myelofibrosis in the bone marrow and spleen. Ts65Dn mice also display a decreased proportion of common lymphoid progenitors associated with a lower level of IL7 receptor expression [92].

The Dp(16); Dp(17); Dp(10) strain is the only model trisomic for all syngeneic regions of human chromosome 21. These mice display macrocytic anemia and also develop myeloproliferative disorder; these phenotypes are conserved in the Dp(16) model alone [93], strongly suggesting that the minimal region implicated in DS-myeloid disorders is contained within Mmu16. In contrast to the Ts65Dn strain, 15-month-old Dp(16); Dp(17); Dp(10) mice have an increased percentage of MEP and a decreased percentage of GMP in the bone marrow. The reason for these differences are not known to date but may result from differences in strain and/or modifier genes.

The Cre/LoxP system has been used to develop trisomy of genes contained in the Down syndrome critical region (DSCR): creating the.... Ts1Rhr model [94]. The DSCR was triplicated on Mmu16 from the CBR1 to FAM3B genes and contains 31 protein coding genes and 2 antisense RNAs [94]. The Ts1Rhr model has been extensively used to assess the role of trisomy of the DSCR in leukemia predisposition and development. Triplication of these 33 regulatory elements alone has no major effect on fetal hematopoiesis, apart from a significant increase in phenotypic HSC. Adult Ts1Rhr mice develop a phenotype similar to the Ts65Dn strain, indicating that trisomy of the DSCR is the minimally required region associated with these myeloid phenotypes [63]. Expression of *Gata1s* in Ts1Rhr led to increased size of CFU-megakaryocyte colonies and transient thrombocytosis mimicking features of TMD. Reproducing the multi-step pathogenesis seen in patient samples by adding a third event in the Ts1Rhr/*Gata1s* model has provided insight on the role of trisomy 21 in TMD/ML–DS development. First, endogenous expression of *JAK3* activating mutations enhance a TMD phenotype during fetal hematopoiesis alone, reinforcing the concept of developmental stage selectivity [95]. Moreover, bone marrow transplantation assays revealed that MPL^W515L^ overexpression functionally cooperates with Gata1s and Ts1Rhr to drive megakaryocytic hyperplasia presenting with phenotypic features of DS–AMKL [63].

The Ts1Rhr strain has also been used to assess the impact of trisomy 21 on B-cell lineage. Compared with wild-type littermates, trisomic mice display a decreased proportion of bone marrow B220+CD43+ early B-cell progenitors, especially Hardy’s fractions B and C (proB cells), and increased clonogenic potential of CFU-preB colonies [74]. Similar to the megakaryocytic lineage, these mice will not spontaneously develop B-cell leukemia and four additional events found in DS–ALL samples (*CRLF2* overexpression, Jak2^R683G^, *Pax5* haploinsufficiency and expression of the dominant negative Ikaros isoform IK6) are required to drive a B-ALL phenotype, although not to full penetrance [74]. As a surrogate, Ts1Rhr was shown to cooperate with p210 BCR-ABL overexpression to develop B-ALL in vivo with shorter latency and complete penetrance, thus demonstrating the impact of trisomy of the DSCR in B-cell leukemogenesis.

In summary, the use of partially trisomic murine models clearly emphasize the role of trisomy 21 in leukemia predisposition and development. While these models can be limited by potential differences between species, they provide the relevant genetic background to study fetal, neonatal and adult hematopoiesis, to investigate the impact of the microenvironment on leukemia predisposition and progression, to identify specific chromosome 21 genes and assess their cooperation with secondary mutations found in human DS-leukemia samples.

### Dosage-sensitive Hsa21 genes and mechanisms altered by trisomy 21 in DS-leukemia

**Fig. 2:**
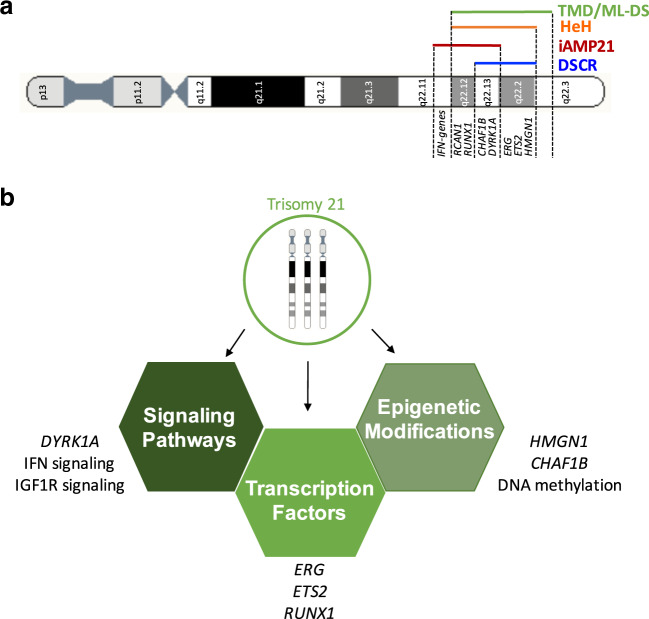
Trisomy 21 and chromosome 21 genes in DS-leukemia. **a** Minimal region of amplification of chromosome 21 in TMD/ML–DS, HeH, iAMP21 and their overlap with the DSCR. **b** Known cellular functions altered by increased dosage of chromosome 21 genes associated with DS-leukemogenesis.

The combination of genetically engineered iPSCs, partially trisomic animal models and characterization of cases with segmental trisomy 21 in patients with DS-leukemia, have been instrumental in identifying dosage-sensitive genes implicated in leukemia predisposition and development in children with DS (Fig. 2 and Table 2) [96, 97].

**Table 2:** List of Hsa21 genes that have a potential role in DS-leukemia.

Hsa21 genes	Known function in hematopoiesis/leukemogenesis	Reference
*ERG*	Promotes megakaryoblastic expansion and cooperates with GATA1s in AMKL	[85, 98–101]
*ETS2*	Cooperates with GATA1s to enhance early hematopoiesis and expansion of fetal megakaryocytic progenitors	[85, 100]
*RUNX1*	Cooperates with ERG, ETS2 and GATA1s to enhance early hematopoiesis	[85]
*DYRK1A*	Promotes TMD/DS–AMKL development in human and murine models; Cooperates with GATA1s to increase megakaryocytic expansion; Controls CFU-preB colony formation and B-cell differentiation	[63, 109]
*RCAN1*	Promotes megakaryopoiesis, Inhibits NFAT pathway	[107]
*HMGN1*	Increases H3K27ac, associated with upregulation of B-cell specific transcriptional signatures	[74, 116]
*CHAF1B*	Interferes with myeloid transcription factor CEBPA and maintains undifferentiated state of leukemic cells	[117]
miR-125b-2	Enhances proliferation and self-renewal of megakaryocytic progenitors, Cooperates with GATA1s	[118]
IFN-genes: *IFNAR1, IFNAR2, IFNGR2* and *IL10RB*	Over-activated in DS blood cells and in fetal hematopoietic progenitors	[81, 110–112]

#### Transcription factors

The chromosome 21 *ERG* oncogene, which encodes a transcription factor from the E-twenty-six (ETS) family, is overexpressed in DS and de novo AMKL [98]. In mice, *ERG* overexpression promotes the expansion of fetal megakaryocytic progenitors, cooperates with *Gata1s* expression and leads to AMKL in vivo [99–101]. Moreover, the loss of one copy of *Erg* (ERG^mld2^ mice) reverts the myeloproliferative phenotype seen in the Ts65Dn model [102]. Possible mechanisms of cooperation between ERG and the secondary alterations found in DS-leukemia include increased chromatin accessibility for ERG (along with RUNX1; another transcription factor encoded by chromosome 21), through the alteration of cohesin complex components [103], and molecular interplay between ERG and the RAS/MAPK pathway, in which ERG induces the transcriptional signature of RAS/MAPK activation and RAS/MAPK regulates ERG activity [104, 105]. Whether this feed-forward loop participates in leukemia development and maintenance in both ML–DS and DS–ALL remains to be investigated. A study using genetically engineered DS-iPSCs suggests that trisomy of *ERG*, together with trisomy of *ETS2* and *RUNX1*, enhances early hematopoiesis and cooperates with *GATA1s* expression [85].

#### Signaling effectors

Trisomy of *DYRK1A* has been shown to promote TMD/DS–AMKL development in human and murine models [63]. *DYRK1A* encodes the dual-specificity tyrosine phosphorylation regulated kinase 1A; a kinase that has multiple targets, thus regulating diverse functions in a cellular context-dependent manner [106]. In murine cells, increased dosage of *Dyrk1a* cooperates with *Gata1s* expression to increase megakaryocytic expansion through inhibition of the calcineurin/NFAT pathway [63]. Another chromosome 21 gene, *RCAN1* (also known as *DSCR1*), encodes a negative regulator of the NFAT pathway, that also contributes to megakaryopoiesis [107]. Since increased dosage of both *DYRK1A* and *RCAN1* cooperate to inhibit neo-angiogenesis in solid tumor development through calcineurin/NFAT pathway inhibition [108], it may be reasonable to assume that a similar additive effect contributes to ML–DS.

Interestingly, *Dyrk1a* has also been shown to regulate B lymphopoiesis [109]. Genetic disruption or pharmacological inhibition of *Dyrk1a* completely abolished CFU-preB colony formation. Dyrk1a also controls the transition between proliferative large preB to quiescent small preB, by triggering cyclin D3 degradation required to exit the cell cycle. Together, these phenotypes indicate that trisomy of *DYRK1A* may promote B-cell leukemia and warrants further investigation.

Over-activation and hypersensitivity to interferon (IFN) signaling, resulting from increased expression of IFN-related genes located on Hsa21 (including *IFNAR1*, *IFNAR2*, *IFNGR2* and *IL10RB*) outside of the DSCR region, has been observed in multiple cell types in individuals with DS [110]. During adulthood, individuals with DS display a perturbed immune system, consistent with a state of chronic inflammation [111, 112]. IFN and inflammatory response transcriptional signatures have been recently observed in murine and human trisomic hematopoietic progenitors, as well as in DS–ALL samples [81] (Laurent A. and Malinge S., unpublished observations), and may be partly mediated by the microenvironment. These observations emphasize the link between IFN signaling, inflammation and immune deficiency in DS. Whether this is implicated in DS–ALL development and reflects the higher rate of B-cell leukemia in children with DS by increasing the risk of infections, which has been suggested as a causal factor for childhood ALL [113, 114], is a promising area for investigation. Moreover, since interferon α signaling has an anti-proliferative effect on DS-associated myeloid disorders in adult bone marrow but not during fetal life [115], this potential interplay between IFN signaling and DS-associated leukemia may also be time and spatially dependent.

#### Epigenetic regulators

*HMGN1* encodes the high mobility group nucleosome-binding protein N1, which modulates accessibility of the histone H3 tail to other epigenetic regulators. Decreased expression or loss of one copy of *Hmgn1* reverts the CFU-preB colonies seen in the Ts1Rhr model, indicative of its key role in leukemia predisposition [74, 116]. Mechanistically, increased dosage of *Hmgn1* increases global H3K27ac and is associated with upregulation of B-cell specific transcriptional signatures [116], which are conserved in DS–ALL and normally associated with a H3K27me3 repressive mark in non-trisomic samples. Another chromatin associated protein encoded by chromosome 21 is CHAF1B, a component of the CAF-1 complex known to drive the first step of nucleosome formation after replication. *CHAF1B* has been shown to be overexpressed in AML, where it maintains the leukemic cells in an undifferentiated state by interfering with the occupancy of the regulator of myeloid differentiation CEBPA [117]. *CHAF1B* is also overexpressed in DS–AMKL [63], but whether it has a similar extra-canonical function in DS-associated leukemia remains elusive to date.

#### Other mechanisms

Chromosome 21 encodes microRNA (miR) and alters several other mechanisms that may impact leukemia development in children with DS. miR-125b-2 is overexpressed in several leukemia subtypes including DS–AMKL and B-ALL, but not in DS–ALL [118, 119]. miR-125b-2 overexpression has been shown to enhance proliferation and self-renewal of megakaryocytic progenitors and synergize with *Gata1s* expression to enhance the DS–AMKL phenotype [118].

Trisomy 21 has also been associated with DNA hypomethylation in TMD/ML–DS, with downregulation of endothelin signaling and over-activity of insulin-like growth factor (IGF) signaling in DS-leukemia primary patient samples, iPSCs and murine models [62, 84, 87, 120], although the chromosome 21 genes and the underlying mechanisms associated with these molecular features remain largely unknown.

## Gain of chromosome 21 in non-DS leukemia

As a somatic event, +21 is one of the most common alterations in hematological cancer (Table 3). Whether findings associated with +21 in DS-leukemia can be extrapolated to other subtypes of leukemia with +21 remains to be seen. Here, we present several subtypes of non-DS leukemia or blood cancer harboring +21, to consider whether studying leukemogenesis in children with DS can have broader application.

**Table 3:** Incidence of gain of chromosome 21 in hematological cancer.

Subtype	Pediatric/Adult	Total cases (*n*)	Total +21 (*n*)	% of +21	Reference
**Myeloid disorders**					
MPN	Adult	938	36	3.8	[144] and MDB
MDS	Adult	3577	151	4.2	[145] and MDB
AML	Pediatric	3758	319	8.5	[133] and MDB
	Adult	17769	695	3.9	[134] and MDB
AMKL	Pediatric	372	126	33.9	[121] and MDB
	Adult	203	15	7.4	[121] and MDB
**Lymphoid disorders**					
T-ALL	Pediatric	1431	43	3.0	[146] and MDB
	Adult	495	30	6.1	MDB
B-ALL	Pediatric	3973	1086	27.3	MDB
	Adult	1290	141	10.9	MDB
CLL	Adult	1432	32	2.2	MDB
FL	Adult	906	87	9.6	[140] and MDB
CTCL	Adult	246	21	8.5	[139, 140] and MDB

*MDB* Mitelman database (updated on 15/10/2019, available at https://mitelmandatabase.isb-cgc.org), *MPN* myeloproliferative neoplasms, *MDS* myelodysplastic syndrome, *AML* acute myeloid leukemia, *AMKL* acute megakaryoblastic leukemia, *T-ALL* T-cell acute lymphoblastic leukemia, *B-ALL* B-cell acute lymphoblastic leukemia, *CLL* chronic lymphocytic leukemia, *FL* follicular lymphoma, *CTCL* cutaneous T-cell lymphoma.

### Gain of chromosome 21 in pediatric non-DS AMKL

Analyses of large cohorts have revealed that +21 is often seen in AMKL, occurring most frequently in children with AMKL [5]. In de novo AMKL, trisomy 21 has been found in all cytogenetically defined subtypes: ETO2-GLIS2, OTT-MAL, NUP98-KDM5A, HOX rearranged and Other [121]. Strikingly, 9.2% of childhood AMKL has a genetic background similar to DS–AMKL (DS–AMKL-*like*: i.e. alterations in genes encoding GATA1, cohesin complex components and signaling effectors together with acquired trisomy 21), with excellent outcome also seen for this subgroup of non-DS children [121]. DS–AMKL-*like* development has been reported in a patient with Cornelia de Lange syndrome, characterized by a mutation in *NIPBL* (encoding a cohesin complex component), with acquired trisomy 21, a *GATA1* mutation, along with EZH2 and JAK/RAS alterations [122]. Moreover, underlying the impact of +21 in TMD development, rare TMD-*like* cases (occurrence of TMD in children without DS) have been described [123–125]. These studies on TMD/DS–AMKL-*like* disorders not only emphasize the cooperative role of trisomy 21, but also raise the question regarding the order of acquisition of these somatic events. Whether trisomy 21 is a founder alteration that is required to ‘prime’ the cellular context susceptible for *GATA1s* expression as highlighted in DS–TMD, or whether +21 cooperates with other genetic alterations in non-DS leukemia, regardless of whether it is an early or late event, remains unknown.

### Gain of chromosome 21 in pediatric B-ALL

B-ALL is the most common type of childhood cancer, and accounts for approximately two-thirds of all pediatric acute leukemia. Complete or partial gain of chromosome 21 is one of the most frequent chromosomal alterations in childhood B-ALL, found in nearly 30% of cases compared with ~11% in adults. It is predominantly seen in HeH, which comprises 25–30% of all pediatric B-ALL, where more than 90% of cases harbor between one and three additional copies of whole chromosome 21, with the majority being tetrasomic [126]. Intra-chromosomal amplification of chromosome 21 (iAMP21) occurs in 2% of pediatric B-ALL. iAMP21 is characterized by a rearranged chromosome 21 patterned with amplified and deleted genomic regions [127, 128]. As in DS–ALL, there is a high incidence of genetic alterations affecting signaling effectors (NRAS, KRAS, FLT3, and SH2B3) in HeH and iAMP21 subtypes [129–131]. However, the molecular bases of this possible oncogenic cooperation are currently unknown. The observation that a minimal region of amplification on chromosome 21, found in both HeH and iAMP21 and overlapping with the DSCR [126, 128, 132] (Fig. 2), indicates that increased dosage of specific chromosome 21 genes located in this region, regardless of whether they are amplified by a constitutive or somatic alteration, may play a role in B-cell leukemogenesis.

### Gain of chromosome 21 in other hematological malignancies

Trisomy 21 is found in 4–5% of AML overall (8.5% in children and 3.9% in adults) [133, 134] (Table 3). This incidence may be slightly underestimated since focal amplifications of specific regions of chromosome 21, such as band 21q22 that contains the *ERG* oncogene, are also found in AML [135–137]. In human and animal models, *ERG* overexpression leads to the development of lymphoid and myeloid leukemia, and promotes a stem cell and progenitor signature [138, 139]. This may provide a susceptible context for oncogenic cooperation with additional alterations that have been found in primary patient samples (cohesin complex components and RAS/MAPK signaling). Overexpression of the chromosome 21 gene *CHAF1B* has been shown to maintain a stem cell signature in murine AML models [117]. Whether other genes located in the 21q22 region are involved in AML development or maintenance remains unknown.

Complete or partial gain of chromosome 21 is also frequently seen in adult hematological cancers such as lymphoma of B or T-cell origin (Table 3). Trisomy 21 has been shown to be an independent risk factor in follicular lymphoma [140]. Gain of chromosome 21 is seen in 8.5% of cutaneous T-cell lymphoma (CTCL) [141, 142], and its role in CTCL development has been assessed in vivo in cooperation with *JAK3* activating mutations, through establishment of a trisomic murine model of CTCL [95]. However, the chromosome 21 genes involved in these disorders and the associated molecular mechanisms require further investigation.

## Perspective

A significant number of studies have been reported over the last decade. From the clinical aspect, we are now reaching a chemotherapeutic plateau and new therapies are required to further improve outcome for DS–ALL and relapsed ML–DS. Studying the molecular basis of leukemia predisposition and development in children with DS has been instrumental in dissecting the role of trisomy 21 on hematopoiesis, both alone and in cooperation with other genetic events, and led to the identification of several key dosage-sensitive chromosome 21 genes. Integration of fundamental research will provide new actionable targets to improve the outcome for children with DS. However, several important questions remain: Are the same chromosome 21 genes/mechanisms implicated in leukemia predisposition, development and maintenance? Does +21 have a role in response to treatment? Could we therapeutically target chromosome 21 proteins or the mechanisms of cooperation between +21 and somatic alterations? As +21 is frequently seen in non-DS-associated blood cancer, pursuing study of +21 in DS-leukemia may have high translational potential to ultimately provide clinical benefit for many patients with hematological malignancies.
